# Perceived causes of stress among a group of western Canadian dental students

**DOI:** 10.1186/s13104-017-2979-9

**Published:** 2017-12-08

**Authors:** Alyssa Hayes, Jay N. Hoover, Chandima P. Karunanayake, Gerald S. Uswak

**Affiliations:** 1 College of Dentistry, University of Saskatchewan, 105 Wiggins Rd, Saskatoon, SK S7N 5E4 Canada; 2 Canadian Centre for Health and Safety in Agriculture-CCHSA, University of Saskatchewan, Saskatoon, SK S7N 2Z4 Canada

**Keywords:** Stress, Dental education, Dental students

## Abstract

**Objective:**

The demanding nature of dental education, both academically and clinically, results in higher levels of perceived stress among its students. The aim of this study was to determine how dental students at the College of Dentistry, University of Saskatchewan perceived stress. During the 2013–2014 academic year, all students were asked to complete a modified dental environmental survey (DES).

**Results:**

Of the 111 students enrolled at the College that year 92 completed the survey (response rate = 83%). In general, female students reported higher stress levels than males. Higher stress levels were associated with living away from home, concerns about manual dexterity and the transition from pre-clinical to clinical studies. Additionally, students who enter dental school with higher debt loads (> 100,000) report high stress levels relating to finances. This study found that financial and clinical workloads result in high stress levels among dental students.

**Electronic supplementary material:**

The online version of this article (10.1186/s13104-017-2979-9) contains supplementary material, which is available to authorized users.

## Introduction

The Canadian Mental Health Association (CMHA) defines stress as “the body’s response to a real or perceived threat […] [however], most of the threats people face today […] are usually problems that people have to work through” [1]. They go on to state that stress can be both helpful (in terms of motivating people towards problem or task completion) or unhelpful, which is some instances can be associated with problem or task avoidance and physical symptoms (e.g., increased heart rate, sweating, headaches and sleeping difficulties) [1]. Often associated with major life events (i.e., entering a professional school, changing jobs) responses reflect a person’s perception about their ability to handle the situation. Dental education, like other health professional programs, is considered to be demanding both, academically and clinically and is associated with higher levels of stress among students [2]. The literature has shown that stress among dental students is associated with emotional exhaustion, burnout, decreased productivity and lower academic success (lower GPA) [2–4]. Common stressors include marital status, gender, academic year, personality type, clinical training and financial burden [5–7]. Al-Saleh et al. [7] reported that among Saudi dental students, stress levels peaked during the latter years of dental training and were associated with the availability of patients, treatment compliance and supervisor feedback. While there are common stressors (i.e., clinical training, patient treatment and supervisor feedback) identified in the literature, prevalence and impact on student stress levels varies and appears to be related to the teaching institution [6]. In light of these variations and the scarcity of Canadian data on this topic, this study attempted to ascertain sources of perceived stress in undergraduate dental students at the University of Saskatchewan, College of Dentistry.

## Main text

### Materials and methods

All undergraduate dental students enrolled in the Doctor of Dental Medicine (DMD) program at the College of Dentistry, University of Saskatchewan, located in western Canada, were invited to participate in the study. Data collection occurred between January and February 2014, thus allowing for the completion of one semester. This study received ethical approval from the Behaviorial Research Ethics Board (REB), University of Saskatchewan (BEH #12-122). Written informed consent was obtained prior to data collection. Paper-based surveys (see Additional file 1) were administered to all years by a College staff member to ensure anonymity. A modified version of the dental environmental stress (DES) questionnaire, and a 10-item Perceived Stress Scale questionnaire (PSS-10) were used [8]. Both instruments are widely accepted, and have proven to be effective tools to quantify stress levels. Data were collected regarding demographics and motivation for choosing a dental career, analysis was completed using SPSS version 24 (SPSS Inc., Armonk, NY: IBM Corp.). Descriptive statistics were used to describe the study and outcome variables. Internal consistency of the questionnaire was assessed by calculating Cronbach’s alpha. A Kruskal–Wallis test [9] was employed to determine significant differences between year of study, gender, marital status, grade point average, ethnicity, and debt load and debt interest payment. The level of significance was set at p < 0.05.

### Results

The reliability of the questionnaire with all items (49) was 0.959, which indicated significant internal consistency (see Additional file 2). Scale 1 contained 10 items referring to social-related stress (Cronbach’s alpha for internal consistency between the items was 0.802); scale 2 (7 items) dealt with financial related stress (Cronbach’s alpha = 0.902); scale 3 (11 items) related to clinical-related stress (Cronbach’s alpha = 0.851); and the fourth scale (21 items) represented academic-related stress (Cronbach’s alpha = 0.934). The adequacy of items under each of the factors was also assessed by calculating the range of Cronbach’s alpha values.

At the time the study was completed, the College of Dentistry had 111 students enrolled in all years, with a response rate of 83 percent the survey is representative of the student body. The dental student cohort at the College of Dentistry at the time of the study was mostly male (56.5%). Respondents were representative of each academic year of the dental program (see Additional file 3). The majority of respondents were between 23 and 25 years of age (55.4%) and reported being never married or single (66.3%). Of interest is that almost 58 percent reported having a current debt load of greater than $100,000 (CDN).

Table 1 shows that when comparing between each academic years statistical differences in stress levels were seen for: socially related stressors (living away from home); clinical related stressors (concern about manual dexterity and clinical skills, the transition from pre- clinical to clinical studies); and academic related stressors (communication with faculty or staff, fear of failing a course/year, confidence about own decision making, and student’s input into College decision-making) were significantly different (p < 0.05). Among first year dental students, the highest mean DES score was attributed to fear over failing a course or year (mean DES = 4.36; SD 1.07). This level of fear decreased as students progressed through their education, with fourth year students reporting considerably lower levels (mean DES = 2.16, SD 1.71). Additionally, social-related stressors (for all years) such as, a lack of time for relaxation and the lack of holiday time account for mean DES scores of 3.42 and 2.84 respectively. Table 2 looked at the role of gender and marital status on mean DES scores. Female dental students reported higher stress levels (mean DES: female = 1.84; males = 1.21, p < 0.05) for family demands and lack of holiday time (mean DES: females = 3.25; males = 2.51, p < 0.05) when compared to their male counterparts. Dental students who reported being married or common law, also reported higher stress levels than their single counterparts when discussing financial responsibilities, specifically pertaining to tuition costs (mean DES: 3.79 versus 3.06, p < 0.05) and size of current debt load (mean DES: 3.79 versus 2.97, p < 0.05). The role of gender was highlighted with female students reporting statistically higher stress levels pertaining to the social demands of family and the lack of holiday hours compared to their male counterparts (mean DES: 1.84 versus 1.21 and 3.25 versus 2.51 respectively). However, the overall lack of gender differences suggests that gender does not play a large role in student’s stress levels. This trend can also be seen when looking at marital status. Slightly higher levels of stress were reported among students who were married or common law in terms of tuition costs (mean DES: 3.79 versus 3.06) and current debt load (mean DES: 3.79 versus 2.97).

**Table 1:** Mean dental environmental stress (DES) questionnaire scores and comparison among 4 years

Category	Stressor	DES overall	DES by year				Sig. level
			Year 1 (2016)	Year 2 (2015)	Year 3 (2014)	Year 4 (2013)	
		Mean (SD)	Mean (SD)	Mean (SD)	Mean (SD)	Mean (SD)	
Social-related stress	Living away from home	0.92 (1.14)	1.14 (0.96)	1.13 (1.25)	1.00 (1.38)	0.35 (0.61)	0.039*
	Accommodations not being conducive for studying	1.77 (1.59)	2.04 (1.57)	2.00 (1.17)	1.44 (1.63)	1.65 (1.90)	0.292
	Difficulty in making friends	0.62 (0.89)	0.74 (1.10)	0.78 (0.94)	0.56 (0.82)	0.42 (0.61)	0.720
	Romantic relationships	1.63 (1.45)	1.59 (1.26)	1.50 (1.41)	1.81 (1.75)	1.53 (1.31)	0.973
	Lack of time for relaxation	3.42 (1.33)	3.24 (1.13)	3.55 (1.43)	3.81 (1.39)	2.95 (1.27)	0.109
	Lack of holiday time compared to other students	2.84 (1.39)	2.96 (1.10)	2.90 (1.55)	2.63 (1.64)	2.89 (1.24)	0.893
	Social demands-family	1.49 (1.32)	1.18 (1.10)	1.37 (1.07)	1.92 (1.69)	1.39 (1.14)	0.531
	Social demands-friends	1.65 (1.19)	1.80 (0.91)	1.63 (1.12)	1.70 (1.54)	1.39 (1.04)	0.562
	Personal physical health	1.97 (1.62)	1.60 (1.61)	1.60 (1.70)	2.62 (1.79)	1.95 (1.08)	0.088
	Physical health of others-significant other/family	1.51 (1.40)	1.18 (1.05)	1.47 (1.59)	2.14 (1.58)	1.13 (1.15)	0.145
Financial-related stress	Financial responsibilities-living expenses	2.33 (1.50)	2.32 (1.49)	2.05 (1.87)	2.52 (1.45)	2.37 (1.21)	0.757
	Financial responsibilities-disposable income	2.18 (1.58)	2.38 (1.47)	1.71 (1.72)	2.39 (1.67)	2.11 (1.49)	0.499
	Financial responsibilities-tuition costs	3.29 (1.46)	3.42 (1.35)	3.35 (1.49)	3.44 (1.45)	2.84 (1.61)	0.593
	Financial responsibilities-transportation	1.62 (1.28)	1.35 (1.03)	1.33 (1.14)	1.88 (1.37)	1.84 (1.50)	0.404
	Financial responsibilities-size of current debt load	3.23 (1.60)	3.00 (1.62)	3.21 (1.62)	3.42 (1.58)	3.26 (1.69)	0.793
	Financial responsibilities-size of future debt load	3.36 (1.59)	3.42 (1.47)	3.28 (1.60)	3.38 (1.67)	3.32 (1.73)	0.979
	Financial responsibilities-interest payments on debt	2.81 (1.61)	2.50 (1.44)	2.83 (1.65)	3.12 (1.61)	2.74 (1.82)	0.531
Clinical-related stress	Concern about manual dexterity and clinical skills	1.68 (1.39)	2.44 (1.26)	1.40 (1.31)	1.67 (1.49)	1.05 (1.10)	0.003*
	Transition from pre-clinical to clinical studies	1.17 (1.44)	2.68 (1.17)	1.89 (1.66)	3.00 (1.41)	1.90 (1.25)	0.017*
	Completing clinical requirements	2.84 (1.48)	2.93 (1.33)	2.90 (1.33)	3.19 (1.62)	2.21 (1.44)	0.149
	Clinical grading	2.36 (1.35)	3.00 (1.36)	2.05 (1.36)	2.37 (1.33)	2.20 (1.32)	0.236
	Differences in opinions of clinical faculty and staff regarding clinical decision-making and treatment	2.88 (1.39)	3.05 (1.60)	2.75 (1.29)	3.19 (1.36)	2.42 (1.26)	0.237
	Supply of patients	2.86 (1.47)	2.14 (1.21)	2.84 (1.38)	3.33 (1.47)	2.50 (1.54)	0.159
	Patient communication and management	2.27 (1.47)	2.00 (1.15)	2.45 (1.54)	2.33 (1.44)	2.15 (1.63)	0.899
	Confidence in own clinical decision-making	1.77 (1.13)	2.57 (0.94)	1.45 (0.99)	1.81 (1.21)	1.45 (1.05)	0.009*
	Adequacy of clinical supervision	2.04 (1.38)	1.92 (1.31)	1.60 (0.99)	2.41 (1.50)	2.05 (1.54)	0.415
	Patients attending scheduled appointments	2.57 (1.61)	2.00 (1.15)	2.60 (1.82)	2.89 (1.62)	2.30 (1.52)	0.557
	Occupational/health hazards	1.41 (1.37)	1.27 (1.03)	1.15 (1.35)	1.81 (1.52)	1.25 (1.37)	0.292
Academic-related stress	Conducive teaching environment	2.31 (1.37)	1.88 (1.48)	2.79 (1.27)	2.44 (1.28)	2.21 (1.36)	0.100
	Criticism of academic and/or clinical work	2.78 (1.38)	3.04 (1.46)	3.15 (1.35)	2.74 (1.40)	2.00 (1.00)	0.064
	Approachability of faculty/staff	2.16 (1.45)	1.76 (1.45)	2.85 (1.39)	2.15 (1.35)	2.00 (1.53)	0.068
	Communication with faculty/staff	2.27 (1.41)	1.76 (1.42)	2.95 (1.22)	2.41 (1.47)	2.05 (1.27)	0.023*
	Rules/regulations of college	2.62 (1.61)	2.32 (1.75)	2.85 (1.56)	2.89 (1.60)	2.37 (1.50)	0.481
	Expectation versus reality of dental school	3.29 (1.50)	3.40 (1.38)	3.25 (1.71)	3.56 (1.34)	2.79 (1.62)	0.453
	Amount of course work	3.28 (1.30)	3.40 (0.82)	3.20 (1.24)	3.59 (1.39)	2.80 (1.64)	0.306
	Difficulty of course work	2.67 (1.33)	3.08 (0.95)	2.90 (1.52)	2.56 (1.28)	2.10 (1.45)	0.088
	Time available for learning	3.32 (1.31)	3.48 (1.05)	3.55 (1.28)	3.56 (1.28)	2.55 (1.47)	0.051*
	Fear of not being able to catch up if falling behind in course work	3.03 (1.44)	3.28 (1.14)	3.40 (1.43)	3.26 (1.48)	2.00 (1.37)	0.008*
	Fear of failing a course or year	3.68 (1.50)	4.36 (1.07)	4.15 (1.09)	3.78 (1.22)	2.16 (1.71)	< 0.001*
	Competition for grades	1.96 (1.58)	2.36 (1.75)	2.11 (1.52)	1.93 (1.41)	1.35 (1.56)	0.170
	Uncertainty about future dental career	2.01 (1.48)	2.40 (1.58)	2.05 (1.61)	1.74 (1.16)	1.84 (1.61)	0.469
	Examinations	3.05 (1.39)	3.00 (0.98)	3.00 (1.59)	3.56 (1.40)	2.50 (1.47)	0.107
	Lack of input into dental college decision-making	2.48 (1.61)	1.72 (1.49)	3.15 (1.56)	2.68 (1.57)	2.50 (1.57)	0.026*
	Clinical time allotted in curriculum	2.24 (1.32)	2.41 (1.28)	2.40 (1.09)	2.41 (1.55)	1.70 (1.17)	0.176
	The language of teaching	1.03 (1.12)	1.21 (1.21)	1.06 (1.16)	0.96 (1.10)	0.89 (1.05)	0.842
	Knowledge transfer of information, methods and materials	1.63 (1.21)	1.54 (1.18)	1.80 (1.20)	1.69 (1.32)	1.50 (1.19)	0.867
	The amount of material	2.60 (1.41)	2.88 (1.13)	2.45 (1.15)	2.89 (1.60)	2.00 (1.59)	0.108
	The difficulty of material	1.97 (1.20)	2.20 (0.82)	2.05 (1.05)	2.04 (1.48)	1.50 (1.28)	0.152
	Reference and information resources	1.33 (1.17)	1.14 (0.89)	1.32 (1.06)	1.48 (1.42)	1.35 (1.23)	0.934

* Statistically significant at p < 0.05

**Table 2:** Mean dental environmental stress (DES) questionnaire scores and comparison among gender and marital status

Category	Stressor	DES by gender		Sig. level	DES by marital status		Sig. level
		Male	Female		Other	Married/common-law	
		Mean (SD)	Mean (SD)		Mean (SD)	Mean (SD)	
Social-related stress	Living away from home	0.78 (1.01)	1.13 (1.29)	0.216	0.92 (1.08)	0.92 (1.26)	0.781
	Accommodations not being conducive for studying	1.65 (1.52)	1.94 (1.68)	0.451	1.70 (1.55)	1.92 (1.69)	0.633
	Difficulty in making friends	0.56 (0.92)	0.70 (0.84)	0.236	0.63 (0.87)	0.62 (0.94)	0.906
	Romantic relationships	1.80 (1.56)	1.42 (1.31)	0.261	2.14 (1.46)	0.61 (0.74)	< 0.001*
	Lack of time for relaxation	3.33 (1.31)	3.53 (1.36)	0.471	3.35 (1.34)	3.55 (1.30)	0.533
	Lack of holiday time compared to other students	2.51 (1.42)	3.25 (1.26)	0.015*	2.68 (1.43)	3.17 (1.26)	0.127
	Social demands-family	1.21 (1.16)	1.84 (1.44)	0.036*	1.30 (1.26)	1.86 (1.38)	0.055
	Social demands-friends	1.48 (1.15)	1.87 (1.22)	0.158	1.68 (1.20)	1.59 (1.18)	0.677
	Personal physical health	1.74 (1.35)	2.25 (1.89)	0.311	2.11 (1.61)	1.66 (1.63)	0.160
	Physical health of others-significant other/family	1.20 (1.11)	1.91 (1.65)	0.084	1.53 (1.40)	1.46 (1.43)	0.793
Financial-related stress	Financial responsibilities-living expenses	2.20 (1.41)	2.50 (1.61)	0.449	2.12 (1.47)	2.79 (1.47)	0.055
	Financial responsibilities-disposable income	2.10 (1.56)	2.29 (1.62)	0.652	2.02 (1.58)	2.52 (1.55)	0.165
	Financial responsibilities-tuition costs	3.12 (1.45)	3.51 (1.47)	0.178	3.06 (1.52)	3.79 (1.20)	0.035*
	Financial responsibilities-transportation	1.54 (1.27)	1.71 (1.29)	0.535	1.53 (1.18)	1.81 (1.47)	0.510
	Financial responsibilities-size of current debt load	3.15 (1.58)	3.33 (1.64)	0.515	2.97 (1.64)	3.79 (1.40)	0.025*
	Financial responsibilities-size of future debt load	3.23 (1.59)	3.51 (1.60)	0.345	3.14 (1.64)	3.82 (1.39)	0.063
	Financial responsibilities-interest payments on debt	2.70 (1.56)	2.95 (1.68)	0.511	2.58 (1.60)	3.29 (1.56)	0.068
Clinical-related stress	Concern about manual dexterity and clinical skills	1.52 (1.39)	1.90 (1.37)	0.138	1.84 (1.46)	1.37 (1.19)	0.152
	Transition from pre-clinical to clinical studies	2.20 (1.37)	2.76 (1.50)	0.072	2.37 (1.44)	2.57 (1.48)	0.475
	Completing clinical requirements	2.53 (1.53)	3.22 (1.33)	0.059	2.75 (1.44)	3.04 (1.56)	0.386
	Clinical grading	2.20 (1.36)	2.57 (1.33)	0.151	2.40 (1.31)	2.29 (1.46)	0.834
	Differences in opinions of clinical faculty and staff regarding clinical decision-making and treatment	2.71 (1.43)	3.11 (1.33)	0.222	2.88 (1.28)	2.89 (1.62)	0.877
	Supply of patients	2.56 (1.45)	3.21 (1.45)	0.061	2.85 (1.46)	2.88 (1.53)	0.851
	Patient communication and management	2.17 (1.39)	2.40 (1.56)	0.521	2.20 (1.38)	2.41 (1.62)	0.681
	Confidence in own clinical decision-making	1.73 (1.19)	1.81 (1.06)	0.608	1.77 (1.12)	1.75 (1.17)	0.807
	Adequacy of clinical supervision	1.84 (1.46)	2.29 (1.25)	0.065	2.00 (1.37)	2.11 (1.42)	0.661
	Patients attending scheduled appointments	2.15 (1.46)	3.06 (1.67)	0.018*	2.57 (1.64)	2.56 (1.60)	0.973
	Occupational/health hazards	1.25 (1.24)	1.59 (1.50)	0.360	1.42 (1.35)	1.39 (1.42)	0.820
Academic-related stress	Conducive teaching environment	2.12 (1.29)	2.56 (1.45)	0.169	2.13 (1.35)	2.69 (1.36)	0.098
	Criticism of academic and/or clinical work	2.59 (1.34)	3.00 (1.41)	0.185	2.89 (1.38)	2.54 (1.37)	0.230
	Approachability of faculty/staff	2.02 (1.39)	2.35 (1.53)	0.360	2.10 (1.50)	2.30 (1.37)	0.405
	Communication with faculty/staff	2.04 (1.37)	2.55 (1.43)	0.087	2.17 (1.49)	2.47 (1.25)	0.273
	Rules/regulations of College	2.69 (1.59)	2.53 (1.65)	0.616	2.70 (1.59)	2.43 (1.65)	0.447
	Expectation versus reality of dental school	3.08 (1.67)	3.55 (1.22)	0.248	3.16 (1.55)	3.53 (1.38)	0.299
	Amount of course work	3.06 (1.38)	3.58 (1.15)	0.060	3.26 (1.27)	3.33 (1.40)	0.592
	Difficulty of course work	2.50 (1.38)	2.90 (1.24)	0.167	2.55 (1.28)	2.93 (1.41)	0.146
	Time available for learning	3.19 (1.44)	3.48 (1.11)	0.412	3.29 (1.28)	3.37 (1.38)	0.716
	Fear of not being able to catch up if falling behind in course work	2.80 (1.47)	3.33 (1.37)	0.100	3.00 (1.39)	3.10 (1.56)	0.659
	Fear of failing a course or year	3.57 (1.49)	3.83 (1.52)	0.248	3.62 (1.52)	3.80 (1.47)	0.581
	Competition for grades	2.00 (1.53)	1.90 (1.67)	0.650	2.06 (1.50)	1.72 (1.75)	0.206
	Uncertainty about future dental career	1.86 (1.61)	2.21 (1.28)	0.173	1.87 (1.41)	2.31 (1.61)	0.240
	Examinations	2.71 (1.37)	3.50 (1.30)	0.008*	2.95 (1.34)	3.28 (1.51)	0.247
	Lack of input into dental college decision-making	2.22 (1.50)	2.82 (1.70)	0.084	2.33 (1.50)	2.77 (1.79)	0.237
	Clinical time allotted in curriculum	2.26 (1.36)	2.21 (1.30)	0.821	2.18 (1.25)	2.36 (1.47)	0.701
	The language of teaching	1.02 (1.06)	1.05 (1.21)	0.909	1.10 (1.15)	0.89 (1.05)	0.450
	Knowledge transfer of information, methods and materials	1.63 (1.23)	1.64 (1.20)	0.943	1.67 (1.31)	1.55 (0.98)	0.947
	The amount of material	2.38 (1.46)	2.88 (1.32)	0.108	2.68 (1.40)	2.43 (1.45)	0.397
	The difficulty of material	1.83 (1.11)	2.15 (1.29)	0.288	1.95 (1.21)	2.00 (1.20)	0.819
	Reference and information resources	1.28 (1.16)	1.39 (1.20)	0.619	1.32 (1.20)	1.36 (1.13)	0.729

* Statistically significant at p < 0.05

Table 3 illustrates the role of current debt load on student stress levels, significantly higher levels of stress were reported among students incurring a current debt load of greater than $100,000 dollars. This trend was also seen in those reporting a debt related interest payment of greater than $500 dollars.

**Table 3:** Mean dental environmental stress (DES) questionnaire scores and comparison among debt load and debt interest payment

Category	Stressor	DES by debt load		Sig. level	DES by debt interest payment		Sig. level
		≤ $100,000	> $100,000		$0–500	> $500	
		Mean (SD)	Mean (SD)		Mean (SD)	Mean (SD)	
Social-related stress	Living away from home	1.34 (1.32)	0.67 (0.95)	0.008*	0.90 (1.14)	1.00 (1.19)	0.722
	Accommodations not being conducive for studying	1.77 (1.55)	1.81 (1.62)	0.973	1.81 (1.60)	1.71 (1.54)	0.880
	Difficulty in making friends	0.65 (0.85)	0.60 (0.93)	0.654	0.64 (0.94)	0.53 (0.64)	0.947
	Romantic relationships	1.82 (1.53)	1.51 (1.42)	0.310	1.68 (1.45)	1.43 (1.55)	0.460
	Lack of time for relaxation	3.45 (1.31)	3.38 (1.36)	0.863	3.39 (1.35)	3.53 (1.25)	0.763
	Lack of holiday time compared to other students	2.76 (1.34)	2.87 (1.44)	0.745	2.75 (1.40)	3.20 (1.37)	0.291
	Social demands-family	1.17 (1.01)	1.73 (1.48)	0.119	1.32 (1.14)	2.33 (1.80)	0.043*
	Social demands-friends	1.54 (1.12)	1.73 (1.25)	0.608	1.55 (1.12)	2.13 (1.46)	0.117
	Personal physical health	1.92 (1.67)	2.02 (1.62)	0.728	1.84 (1.61)	2.67 (1.59)	0.058
	Physical health of others-significant other/family	1.19 (1.26)	1.83 (1.47)	0.047*	1.41 (1.39)	2.00 (1.36)	0.099
Financial-related stress	Financial responsibilities-living expenses	1.68 (1.32)	2.77 (1.48)	0.001*	2.17 (1.50)	3.13 (1.30)	0.013*
	Financial responsibilities-disposable income	1.68 (1.53)	2.55 (1.54)	0.014*	1.98 (1.57)	3.07 (1.39)	0.011*
	Financial responsibilities-tuition costs	3.00 (1.47)	3.52 (1.43)	0.097	3.24 (1.47)	3.60 (1.45)	0.360
	Financial responsibilities-transportation	1.51 (1.28)	1.73 (1.27)	0.363	1.47 (1.15)	2.40 (1.55)	0.024*
	Financial responsibilities-size of current debt load	2.47 (1.78)	3.77 (1.23)	0.001*	3.07 (1.62)	4.13 (1.12)	0.015*
	Financial responsibilities-size of future debt load	2.76 (1.81)	3.75 (1.33)	0.011*	3.20 (1.63)	4.13 (1.25)	0.030*
	Financial responsibilities-interest payments on debt	2.18 (1.59)	3.25 (1.49)	0.004*	2.59 (1.60)	3.93 (1.16)	0.030*
Clinical-related stress	Concern about manual dexterity and clinical skills	1.82 (1.27)	1.58 (1.49)	0.226	1.79 (1.44)	1.19 (1.11)	0.135
	Transition from pre-clinical to clinical studies	2.49 (1.50)	2.42 (1.42)	0.880	2.52 (1.41)	2.13 (1.59)	0.227
	Completing clinical requirements	2.68 (1.25)	2.98 (1.60)	0.294	2.89 (1.38)	2.75 (1.84)	0.811
	Clinical grading	2.23 (1.19)	2.44 (1.46)	0.573	2.31 (1.26)	2.56 (1.75)	0.665
	Differences in opinions of clinical faculty and staff regarding clinical decision-making and treatment	2.82 (1.45)	2.94 (1.38)	0.800	2.91 (1.40)	2.81 (1.42)	0.859
	Supply of patients	2.96 (1.45)	2.80 (1.50)	0.662	2.90 (1.40)	2.73 (1.79)	0.797
	Patient communication and management	2.79 (1.52)	1.98 (1.36)	0.022*	2.34 (1.48)	2.00 (1.41)	0.401
	Confidence in own clinical decision-making	2.00 (1.34)	1.64 (0.98)	0.286	1.82 (1.16)	1.60 (1.06)	0.757
	Adequacy of clinical supervision	2.00 (1.49)	2.08 (1.33)	0.703	2.11 (1.38)	1.80 (1.42)	0.367
	Patients attending scheduled appointments	2.68 (1.72)	2.50 (1.56)	0.609	2.61 (1.64)	2.40 (1.55)	0.661
	Occupational/health hazards	1.57 (1.52)	1.32 (1.28)	0.616	1.50 (1.41)	1.06 (1.18)	0.246
Academic-related stress	Conducive teaching environment	2.08 (1.34)	2.48 (1.39)	0.173	2.30 (1.42)	2.40 (1.18)	0.563
	Criticism of academic and/or clinical work	2.92 (1.40)	2.66 (1.38)	0.399	2.84 (1.41)	2.38 (1.19)	0.267
	Approachability of faculty/staff	1.87 (1.38)	2.37 (1.49)	0.148	2.12 (1.47)	2.31 (1.45)	0.604
	Communication with faculty/staff	1.95 (1.45)	2.50 (1.36)	0.089	2.27 (1.45)	2.25 (1.29)	0.952
	Rules/regulations of College	2.16 (1.46)	2.94 (1.66)	0.031*	2.58 (1.58)	2.75 (1.84)	0.796
	Expectation versus reality of dental school	2.95 (1.47)	3.50 (1.49)	0.070	3.22 (1.48)	3.50 (1.59)	0.430
	Amount of course work	3.34 (1.12)	3.25 (1.44)	0.964	3.32 (1.23)	3.13 (1.67)	0.855
	Difficulty of course work	2.87 (1.23)	2.53 (1.39)	0.276	2.68 (1.29)	2.63 (1.59)	0.957
	Time available for learning	3.53 (1.25)	3.17 (1.35)	0.219	3.43 (1.25)	2.81 (1.51)	0.136
	Fear of not being able to catch up if falling behind in course work	3.16 (1.40)	2.94 (1.49)	0.536	3.11 (1.38)	2.69 (1.74)	0.421
	Fear of failing a course or year	3.79 (1.28)	3.58 (1.65)	0.898	3.74 (1.45)	3.31 (1.70)	0.285
	Competition for grades	2.00 (1.49)	1.94 (1.67)	0.786	1.95 (1.58)	2.06 (1.69)	0.809
	Uncertainty about future dental career	1.76 (1.38)	2.14 (1.49)	0.250	1.92 (1.41)	2.25 (1.65)	0.475
	Examinations	2.95 (1.35)	3.12 (1.44)	0.494	3.11 (1.35)	2.75 (1.61)	0.453
	Lack of input into dental college decision-making	2.00 (1.45)	2.85 (1.64)	0.016*	2.51 (1.63)	2.44 (1.55)	0.957
	Clinical time allotted in curriculum	2.36 (1.19)	2.18 (1.41)	0.448	2.18 (1.28)	2.60 (1.50)	0.302
	The language of teaching	1.00 (1.09)	1.08 (1.15)	0.761	0.97 (1.14)	1.38 (1.02)	0.097
	Knowledge transfer of information, methods and materials	1.59 (1.17)	1.67 (1.26)	0.850	1.60 (1.19)	1.81 (1.38)	0.616
	The amount of material	2.82 (1.25)	2.18 (1.09)	0.161	2.64 (1.36)	2.44 (1.71)	0.479
	The difficulty of material	2.18 (1.09)	1.81 (1.27)	0.071	1.93 (1.13)	2.13 (1.54)	0.842
	Reference and information resources	1.56 (1.34)	1.20 (1.02)	0.247	1.32 (1.19)	1.44 (1.09)	0.563

* Statistically significant at p < 0.05

### Discussion

This study presents current data on the role of student stress in dental education, and importantly presents data from western Canada, specifically the prairies. As the survey was conducted at the College of Dentistry, University of Saskatchewn the results cannot be generalized to all Canadian dental schools. High stress levels reported due to lack of time for relaxation among students at the University of Saskatchewan (means DES = 3.42) is consistent with Muirhead [10], who reported a mean DES of 3.14 for students at another Canadian dental school. Contextually, the University of Saskatchewan, begins classes in mid-August and continues until April or May (depending on year of program), the longer academic term coupled with the understanding that dental education places demands above and beyond the normal school hours, the findings are consistent with the literature [11]. Also consistent with the literature is the finding that clinical-related stressors, in particular those dealing with the transition from pre-clinical to clinical work, (i.e., clinical grading, patient supply and communication) are highest among 3rd year dental students (mean DES ranges from 2.33 to 3.33). Thus re-iterating the belief that the aforementioned transition marks an integral and stressful time in a student’s dental education [12, 13]. High levels of stress were associated with differing opinions of clinical faculty and staff regarding decision making and treatment (mean DES scores ranged from 3.05 in 1st year to 2.42 in 4th year). Clinical faculty is largely comprised of practicing or newly retired clinicians willing to provide their time, and thus bring differing experiences and backgrounds [14]. Currently, the College of Dentistry has no formalized program to calibrate clinical faculty, as some other dental institutions do. The high levels of stress attributed to this lack of clarity from faculty highlights the need for a calibration program to be implemented, potentially mitigating some stress levels among the students. Additionally, the incorporation of stress management strategies (i.e., mindfulness, therapy dogs) during high stress times (e.g., examination periods) should be explored by dental educators.

This study found that when looking at financial-related stressors, significant differences were seen between those with smaller debt loads (≤ $100,000) and those carrying larger debt loads (≥ $100,000), in all categories except tuition costs. Of interest is that higher stress due to living away from home was seen among those with lower debt loads (mean DES score of 1.34) compared to those with larger debts, while students living with their parents had significantly higher dental school entry debts. Similar findings were reported among Canadian dental students in Toronto, Canada [10]. This is important as the Financial Consumer Agency of Canada reported that tuition for the 2013–14 academic year rose 3.3 percent over the previous year and was expected to increase further from the reported Canadian average of $5772.00 [15]. When looking at the costs of dental education from 2010/11 to 2014/15 average tuition costs rose from $15,062.00 to $18,187.00 per year [16]. It is not surprising then, that students with higher existing debt loads and interest payments had significantly higher self-reported stress levels relating to finances. Discussions around debt loads and costs of tuition are important, especially when post-secondary institutions are faced with increased operating costs and funding reductions.

### Conclusions

The findings of this study are consistent with the literature, which states that financial and clinical workloads result in high stress levels among dental students. Dental educators must be cognizant of their responsibility to ensure that students, especially at the beginning of their education, have realistic expectations pertaining to issues such as workloads and costs of education. Of equal importance, all faculty especially clinical instructors, should be educated on grading systems and calibrated to help mitigate student stress as it pertains to faculty-student interactions. From a policy perspective, a formalized onboarding program for faculty and staff is recommended prior to interacting with students.

## Limitations

This study was conducted at a single Canadian dental education institution (University of Saskatchewan) and the results cannot be generalized to other Canadian dental education institutions. However, the data was representative of the students enrolled at the College of Dentistry as evidenced by the high response rate. The findings represent the first time data on student stress was reported for a dental education institution located in western Canada thus providing insight for educators into student stress levels. Further research is needed to determine if similar results would be reported by dental students across Canada.

## Additional files

**Additional file 1.** Dental Environment Stress Survey.

**Additional file 2.** Reliability of stress factors (subscales) of DES Questionnaire.

**Additional file 3.** Socio-demographic characteristics of the participants.

## Abbreviations

DES: dental environment survey
CMHA: Canadian Mental Health Association
GPA: grade point average
DMD: Doctor of Medicine in Dentistry
PSS-10: Perceived Stress Scale 10
SPSS: statistical Package for the Social Sciences
