# Supplementary material for: Perceived causes of stress among a group of western Canadian dental students
Source: BMC Res Notes. 2017 Dec 8;10:714. doi: 10.1186/s13104-017-2979-9 (PMC5721618; doi:10.1186/s13104-017-2979-9)
Supplement: Supplementary file 1 — Additional file 1. Dental Environment Stress Survey. [file 13104_2017_2979_MOESM1_ESM.doc]

# Dental Environment Stress Survey

These questions are designed to assess the type of stressful situations dental students face throughout their time in dental school

1. Read each item carefully
2. Think about how often you have experienced the behavior, attitude or policy described in the item
3. Please respond to every item
4. Determine your response by choosing one of the categories of frequency given below. Choose the category that most closely approximates your perceptions using the following rating scale:

| Not  Stressful  At All  0 | Slightly Stressful  1 | Fairly Stressful  2 | Very  Stressful  3 | Highly Stressful  4 | Extremely Stressful  5 | Not  Applicable  6 |
| --- | --- | --- | --- | --- | --- | --- |

# Please indicate the stressfulness of the following situations:

| Not  Stressful  At All  0 | Slightly Stressful  1 | Fairly  Stressful  2 | Very  Stressful  3 | Highly  Stressful  4 | Extremely Stressful  5 | Not Applicable  6 |
| --- | --- | --- | --- | --- | --- | --- |

|  |  | | | | | | | | | | | | | | |
| --- | --- | --- | --- | --- | --- | --- | --- | --- | --- | --- | --- | --- | --- | --- | --- |
| Living Accommodations | | | | | | | | | | | | | | |  |
|  | |  | | | | | | | | | | | | |  |
| 1. Living away from home | | a | | b | | c | | d | | e | f | | g | |  |
| 1. Accommodations not being conducive for studying | | a | | b | | c | | d | | e | f | | g | |  |
| 1. Living with friends or roommates | | a | | b | | c | | d | | e | f | | g | |  |
| 1. Living with significant others | | a | | b | | c | | d | | e | f | | g | |  |
| 1. Living with family | | a | | b | | c | | d | | e | f | | g | |  |
| 1. Living with children | | a | | b | | c | | d | | e | f | | g | |  |
| 1. Accommodations lack recreation/leisure facilities | | a | | b | | c | | d | | e | f | | g | |  |
| 1. Cost of accommodations – rent or mortgage | | a | | b | | c | | d | | e | f | | g | |  |
| Please Proceed to Next Page | | | | | | | | | | | | | | |  |
|  | | | | | | | | | | | | | | |  |
| Personal Factors | | | | | | | | | | | | | | |  |
|  | | | | | | | | | | | | | | |  |
| 1. Difficulty in making friends | a | | b | | c | | d | | e | | | f | | g | |
| 1. Romantic relationships | a | | b | | c | | d | | e | | | f | | g | |
| 1. Lack of time for relaxation | a | | b | | c | | d | | e | | | f | | g | |
| 1. Lack of holiday time compared to other students | a | | b | | c | | d | | e | | | f | | g | |
| 1. Social Demands – spouse or significant other | a | | b | | c | | d | | e | | | f | | g | |
| 1. Social Demands – Family | a | | b | | c | | d | | e | | | f | | g | |
| 1. Social Demands – Friends | a | | b | | c | | d | | e | | | f | | g | |
| 1. Conflicts with significant other regarding career | a | | b | | c | | d | | e | | | f | | g | |
| 1. Conflicts with significant other regarding children | a | | b | | c | | d | | e | | | f | | g | |
| 1. Conflicts with significant other regarding finances | a | | b | | c | | d | | e | | | f | | g | |
| 1. Having to postpone marriage | a | | b | | c | | d | | e | | | f | | g | |
| 1. Having to postpone having children | a | | b | | c | | d | | e | | | f | | g | |
| 1. Having multiple roles | a | | b | | c | | d | | e | | | f | | g | |
| 1. Personal physical health | a | | b | | c | | d | | e | | | f | | g | |
| 1. Physical health of others - significant other/family | a | | b | | c | | d | | e | | | f | | g | |
| 1. Financial responsibilities – living expenses | a | | b | | c | | d | | e | | | f | | g | |
| 1. Financial responsibilities – disposable income | a | | b | | c | | d | | e | | | f | | g | |
|  | Not  Stressful  At All  0 | | Slightly Stressful  1 | | Fairly  Stressful  2 | | Very  Stressful  3 | | Highly  Stressful  4 | | | Extremely Stressful  5 | | Not Applicable  6 | |
| 1. Financial responsibilities – tuition costs | a | | b | | c | | d | | e | | | f | | g | |
| 1. Financial responsibilities –   Transportation | a | | b | | c | | d | | e | | | f | | g | |
| 1. Financial responsibilities – size of current debt load | a | | b | | c | | d | | e | | | f | | g | |
| 1. Financial responsibilities – size of future debt load | a | | b | | c | | d | | e | | | f | | g | |
| 1. Financial responsibilities – interest payments on debt | a | | b | | c | | d | | e | | | f | | g | |
| 1. Behaviour – smoking | a | | b | | c | | d | | e | | | f | | g | |
| 1. Behaviour – Alcohol consumption | a | | b | | c | | d | | e | | | f | | g | |
| 1. Behaviour – Recreational/prescription drugs | a | | b | | c | | d | | e | | | f | | g | |
|  | | | | | | | | | | | | | | |  |
| **Educational Environment – Academic & Clinical** | | | | | | | | | | | | | | |  |
|  | | | | | | | | | | | | | | |  |
|  | Not  Stressful  At All  0 | | Slightly Stressful  1 | | Fairly  Stressful  2 | | Very  Stressful  3 | | Highly  Stressful  4 | | | Extremely Stressful  5 | | Not Applicable  6 | |
| 1. Conducive teaching environment | a | | b | | c | | d | | e | | | f | | g | |
| 1. Criticism of academic and/or clinical work | a | | b | | c | | d | | e | | | f | | g | |
| 1. Approachability of faculty/staff | a | | b | | c | | d | | e | | | f | | g | |
| 1. Communication with faculty/staff | a | | b | | c | | d | | e | | | f | | g | |
| 1. Rules/regulations of College | a | | b | | c | | d | | e | | | f | | g | |
| 1. Expectation versus reality of dental school | a | | b | | c | | d | | e | | | f | | g | |
|  | Not  Stressful  At All  0 | | Slightly Stressful  1 | | Fairly  Stressful  2 | | Very  Stressful  3 | | Highly  Stressful  4 | | | Extremely Stressful  5 | | Not Applicable  6 | |
| 1. Discrimination due to race, nationality, gender or social class | a | | b | | c | | d | | e | | | f | | g | |
| 1. Amount of coursework | a | | b | | c | | d | | e | | | f | | g | |
| 1. Difficulty of coursework | a | | b | | c | | d | | e | | | f | | g | |
| 1. Time available for learning | a | | b | | c | | d | | e | | | f | | g | |
| 1. Fear of not being able to catch up if falling behind in coursework | a | | b | | c | | d | | e | | | f | | g | |
| 1. Fear of failing a course or year | a | | b | | c | | d | | e | | | f | | g | |
| 1. Competition for grades | a | | b | | c | | d | | e | | | f | | g | |
| 1. Uncertainty about future dental career | a | | b | | c | | d | | e | | | f | | g | |
| 1. Examinations | a | | b | | c | | d | | e | | | f | | g | |
| 1. Lack of input into dental college decision-making | a | | b | | c | | d | | e | | | f | | g | |
| 1. Concern about manual dexterity and clinical skills | a | | b | | c | | d | | e | | | f | | g | |
| 1. Transition from pre-clinical to clinical studies | a | | b | | c | | d | | e | | | f | | g | |
| 1. Completing clinical requirements | a | | b | | c | | d | | e | | | f | | g | |
| 1. Clinical grading | a | | b | | c | | d | | e | | | f | | g | |
| 1. Differences in opinions of clinical faculty and staff regarding clinical decision-making and treatment | a | | b | | c | | d | | e | | | f | | g | |
| 1. Clinical time allotted in curriculum | a | | b | | c | | d | | e | | | f | | g | |
|  | Not  Stressful  At All  0 | | Slightly Stressful  1 | | Fairly  Stressful  2 | | Very  Stressful  3 | | Highly  Stressful  4 | | | Extremely Stressful  5 | | Not Applicable  6 | |
| 1. Supply of patients | a | | b | | c | | d | | e | | | f | | g | |
| 1. Patient communication and management | a | | b | | c | | d | | e | | | f | | g | |
| 1. Confidence in own clinical decision-making | a | | b | | c | | d | | e | | | f | | g | |
| 1. Adequacy of clinical supervision | a | | b | | c | | d | | e | | | f | | g | |
| 1. The language of teaching | a | | b | | c | | d | | e | | | f | | g | |
| 1. Knowledge transfer of information, methods and materials | a | | b | | c | | d | | e | | | f | | g | |
| 1. The amount of material | a | | b | | c | | d | | e | | | f | | g | |
| 1. The difficulty of material | a | | b | | c | | d | | e | | | f | | g | |
| 1. Reference and information resources | a | | b | | c | | d | | e | | | f | | g | |
| 1. Supply of patients | a | | b | | c | | d | | e | | | f | | g | |
| 1. Patient attending scheduled appointments | a | | b | | c | | d | | e | | | f | | g | |
| 1. Occupational/health hazards | a | | b | | c | | d | | e | | | f | | g | |

| Not  Stressful  At All  0 | Slightly Stressful  1 | Fairly  Stressful  2 | Very  Stressful  3 | Highly  Stressful  4 | Extremely Stressful  5 | Not Applicable  6 |
| --- | --- | --- | --- | --- | --- | --- |

**Perceived Stress Scale**

The questions in this scale ask you about your feelings and thoughts during the **last month.** In each case, you will be asked to indicate by circling how often you felt or thought a certain way.

**a = Never, b = Almost Never, c = Sometimes, d = Fairly Often e = Very Often**

1. In the last month, how often have you been upset because of something that happened unexpectedly?
2. In the last month, how often have you felt that you were unable to control the important things in your life?
3. In the last month, how often have you felt nervous and “stressed”?
4. In the last month, how often have you felt confident about your ability to handle your personal problems?
5. In the last month, how often have you felt that things were going your way?
6. In the last month, how often have you found that you could not cope with all the things that you had to do?
7. In the last month, how often have you been able to control irritations in your life?
8. In the last month, how often have you felt that you were on top of things?
9. In the last month, how often have you been angered because of things that were outside of your control?
10. In the last month, how often have you felt difficulties were piling up so high that you could not overcome them?
