# Supplementary material for: Perceived causes of stress among a group of western Canadian dental students
Source: BMC Res Notes. 2017 Dec 8;10:714. doi: 10.1186/s13104-017-2979-9 (PMC5721618; doi:10.1186/s13104-017-2979-9)
Supplement: Supplementary file 2 — Additional file 2. Reliability of stress factors (subscales) of DES Questionnaire. [file 13104_2017_2979_MOESM2_ESM.docx]

Reliability of stress factors (subscales) of DES Questionnaire

| **Factor (item numbers)** | **No. items** | **Cronbach’s alpha** | **Cronbach’s alpha if items deleted (range)** |
| --- | --- | --- | --- |
| Social-related stress  (146, 147, 154, 155, 156, 157, 159, 160, 167, 168) | 10 | 0.802 | (0.762 - 0.821) |
| Financial-related stress  (169, 170, 171, 172, 173, 174, 175) | 7 | 0.902 | (0.871 - 0.920) |
| Clinical-related stress  (199, 200, 201, 202, 203, 205, 206, 207, 208, 216, 217) | 11 | 0.851 | (0.827 – 0.853) |
| Academic-related stress  (179, 180, 181, 182, 183, 184, 186, 187, 188, 189, 190, 191, 192, 197, 198, 204, 209, 210, 212, 213, 214) | 21 | 0.934 | (0.929 – 0.934) |
|  |  |  |  |
| All items | 49 | 0.959 | (0.956 – 0.960) |
