# Supplementary material for: Perceived causes of stress among a group of western Canadian dental students
Source: BMC Res Notes. 2017 Dec 8;10:714. doi: 10.1186/s13104-017-2979-9 (PMC5721618; doi:10.1186/s13104-017-2979-9)
Supplement: Supplementary file 3 — Additional file 3. Socio-demographic characteristics of the participants. [file 13104_2017_2979_MOESM3_ESM.docx]

Socio-demographic characteristics of the participants (N=92)

| **Variable** | **n (%)** |
| --- | --- |
| Gender |  |
| Male | 52 (56.5) |
| Female | 40 (43.5) |
| Study Year |  |
| Year 1 (2016) | 25 (27.2) |
| Year 2 (2015) | 20 (21.7) |
| Year 3 (2014) | 27 (29.3) |
| Year 4 (2013) | 20 (21.7) |
| Age group, in years |  |
| 20-22 | 14 (15.2) |
| 23-25 | 51 (55.4) |
| 26-28 | 17 (18.5) |
| 29-31 | 8 (8.7) |
| 32-34 | 2 (2.2) |
| Marital Status |  |
| Never married-single | 61 (66.3) |
| Legally married and not separated | 21 (22.8) |
| Common-law relationships | 9 (9.8) |
| Widowed | 1 (1.1) |
| Ethnic Background |  |
| Caucasian | 68 (73.9) |
| Non-Caucasian | 24 (26.1) |
| Grade Point Average Prior to Applying to Dental School |  |
| ≤ 88 | 34 (37.0) |
| >88 | 57 (62.0) |
| Missing | 1 (1.1) |
| Debt Load |  |
| ≤ $100,000 | 38 (41.3) |
| >$100,000 | 53 (57.6) |
| Missing | 1 (1.1) |
| Debt Interest  $0- $500 per month | 75 (81.5) |
| >$500 per month | 16 (17.4) |
| Missing | 1 (1.1) |
